# Speeding up the Consensus Clustering methodology for microarray data analysis

**DOI:** 10.1186/1748-7188-6-1

**Published:** 2011-01-14

**Authors:** Raffaele Giancarlo, Filippo Utro

**Affiliations:** 1 Dipartimento di Matematica ed Informatica, Universitá di Palermo, Via Archirafi 34, 90123 Palermo, Italy; 2 IBM T.J. Watson Research Center, Yorktown Yeights, NY, USA

## Abstract

**Background:**

The inference of the number of clusters in a dataset, a fundamental problem in Statistics, Data Analysis and Classification, is usually addressed via internal validation measures. The stated problem is quite difficult, in particular for microarrays, since the inferred prediction must be sensible enough to capture the inherent biological structure in a dataset, e.g., functionally related genes. Despite the rich literature present in that area, the identification of an internal validation measure that is both fast and precise has proved to be elusive. In order to partially fill this gap, we propose a speed-up of Consensus (Consensus Clustering), a methodology whose purpose is the provision of a prediction of the number of clusters in a dataset, together with a dissimilarity matrix (the consensus matrix) that can be used by clustering algorithms. As detailed in the remainder of the paper, Consensus is a natural candidate for a speed-up.

**Results:**

Since the time-precision performance of Consensus depends on two parameters, our first task is to show that a simple adjustment of the parameters is not enough to obtain a good precision-time trade-off. Our second task is to provide a fast approximation algorithm for Consensus. That is, the closely related algorithm FC (Fast Consensus) that would have the same precision as Consensus with a substantially better time performance. The performance of FC has been assessed via extensive experiments on twelve benchmark datasets that summarize key features of microarray applications, such as cancer studies, gene expression with up and down patterns, and a full spectrum of dimensionality up to over a thousand. Based on their outcome, compared with previous benchmarking results available in the literature, FC turns out to be among the fastest internal validation methods, while retaining the same outstanding precision of Consensus. Moreover, it also provides a consensus matrix that can be used as a dissimilarity matrix, guaranteeing the same performance as the corresponding matrix produced by Consensus. We have also experimented with the use of Consensus and FC in conjunction with NMF (Nonnegative Matrix Factorization), in order to identify the correct number of clusters in a dataset. Although NMF is an increasingly popular technique for biological data mining, our results are somewhat disappointing and complement quite well the state of the art about NMF, shedding further light on its merits and limitations.

**Conclusions:**

In summary, FC with a parameter setting that makes it robust with respect to small and medium-sized datasets, i.e, number of items to cluster in the hundreds and number of conditions up to a thousand, seems to be the internal validation measure of choice. Moreover, the technique we have developed here can be used in other contexts, in particular for the speed-up of stability-based validation measures.

## Background

Microarray technology for profiling gene expression levels is a popular tool in modern biological research. It is usually complemented by statistical procedures that support the various stages of the data analysis process [1]. Since one of the fundamental aspects of the technology is its ability to infer relations among the hundreds (or even thousands) of elements that are subject to simultaneous measurements via a single experiment, cluster analysis is central to the data analysis process: in particular, the design of (i) new clustering algorithms and (ii) new internal validation measures that should assess the biological relevance of the clustering solutions found. Although both of those topics are widely studied in the general data mining literature, e.g., [2-9], microarrays provide new challenges due to the high dimensionality and noise levels of the data generated from any single experiment. However, as pointed out by Handl et al. [10], the bioinformatics literature has given prominence to clustering algorithms, e.g., [11], rather than to validation procedures. Indeed, the excellent survey by Handl et al. is a big step forward in making the study of those validation techniques a central part of both research and practice in bioinformatics, since it provides both a technical presentation as well as valuable general guidelines about their use for post-genomic data analysis. Although much remains to be done, it is, nevertheless, an initial step.

Based on the above considerations, this paper focuses on data-driven internal validation measures, particularly on those designed for and tested on microarray data. That class of measures assumes nothing about the structure of the dataset, which is inferred directly from the data.

In the general data mining literature, there is a great proliferation of research on clustering algorithms, in particular for gene expression data [12]. Some of those studies concentrate both on the ability of an algorithm to obtain a high quality partition of the data and on its performance in terms of computational resources, mainly CPU time. For instance, hierarchical clustering and K-means algorithms [13] have been the object of several speed-ups (see [14-16] and references therein). Moreover, the need for computational performance is so acute in the area of clustering for microarray data that implementations of well known algorithms, such as K-means, specific for multi-core architectures are being proposed [17]. As far as validation measures are concerned, there are also several general studies, e.g., [18], aimed at establishing the intrinsic, as well as the relative, merit of a measure. However, for the special case of microarray data, the experimental assessment of the "fitness" of a measure has been rather ad hoc and studies in that area provide only partial comparison among measures, e.g., [19]. Moreover, contrary to research in the clustering literature, the performance of validation methods in terms of computational resources, again mainly CPU time, is hardly assessed both in absolute and relative terms.

In order to partially fill the gap existing between the general data analysis literature and the special case of microarray data, Giancarlo et al. [20] have recently proposed an extensive comparative analysis of validation measures taken from the most relevant paradigms in the area: (a) hypothesis testing in statistics, e.g., [21]; (b) stability-based techniques, e.g., [19,22,23] and (c) jackknife techniques, e.g., [24]. These benchmarks consider both the ability of a measure to predict the correct number of clusters in a dataset and, departing from the current state of the art in that area, the computer time it takes for a measure to complete its task. Since the findings of that study are essential to place this research in a proper context, we highlight them next:

(A) There is a very natural hierarchy of internal validation measures, with the fastest and less precise at the top. In terms of time, there is a gap of at least two orders of magnitude between the fastest, WCSS[6], and the slowest ones.

(B) All measures considered in that study have severe limitations on large datasets with a large number of clusters, either in their ability to predict the correct number of clusters or to finish their execution in a reasonable amount of time, e.g, a few days.

(C) Although among the slowest, Consensus[19] displays some quite remarkable properties that, accounting for (A) and (B), make it the measure of choice for small and medium sized datasets. Indeed, it is very reliable in terms of its ability to predict the correct number of clusters in a dataset, in particular when used in conjunction with hierarchical clustering algorithms. Moreover, such a performance is stable across the choice of basic clustering algorithms, i.e., various versions of hierarchical clustering and K-means, used to produce clustering solutions.

It is also useful to recall that, prior to the study of Giancarlo et al., Consensus was already a reference point in the area of internal validation measures, as we outline next.

(D) Monti et. al. [19] had already established the excellence of the Consensus methodology via a comparison with the Gap Statistics [21]. In view of that paper, the contribution by Giancarlo et al. is to give indication of such an excellence with respect to a wider set of measures, showing also its computational limitations. Moreover, Monti et al. also showed that the methodology can be used to obtain dissimilarity matrices that seem to improve the performance of clustering algorithms, in particular hierarchical ones. Additional remarkable properties of that methodology, mainly its ability to discover "natural hierarchical structure" in microarray data, have been highlighted by Brunet et al. [25] in conjunction with NMF, a very versatile pattern discovery technique that has received quite a bit of attention in the computational biology literature, as discussed in the review by Devarajan [26].

(E) Some of the ideas and techniques involved in the Consensus methodology are of a fundamental nature and quite ubiquitous in the cluster validation area. We limit ourselves mentioning that they appear in work prior to that of Monti et al. for the assessment of cluster quality for microarray data analysis [27] and that there are stability-based internal validation methods, i.e., [22,23,28-31], that use essentially the same algorithmic strategy of Consensus to collect information about the structure present in the input dataset, as briefly detailed in the Methods section.

One open question that was made explicit by the study of Giancarlo et al. is the design of a data-driven internal validation measure that is both precise and fast, and capable of granting scalability with dataset size. Such a lack of scalability for the most precise internal validation measures is one of the main computational bottlenecks in the process of cluster evaluation for microarray data analysis. Its elimination is far from trivial [32] and even partial progress on this problem is perceived as important.

Based on its excellent performance and paradigmatic nature, Consensus is a natural candidate for the investigation of an algorithmic speed-up aimed at reducing the mentioned bottleneck. To this end, here we propose FC, which is a fast approximation of Consensus. Indeed, we show, experimentally, that FC is at least an order of magnitude faster than Consensus when used in conjunction with hierarchical clustering algorithms or partitional algorithms with a hierarchical initialization. As discussed in the Conclusions section, the net effect is a substantial reduction in the time gap existing between the fastest measures, i.e., WCSS, and the most precise ones, i.e., Consensus and FC. Moreover, based on our study, several conclusions of methodological value are also offered. In the remainder of this paper, we concentrate on Consensus and FC as internal validation measures. The part regarding their ability to produce good dissimilarity matrices that can be used by clustering algorithms is presented in the Supplementary File at the supplementary material web site [33].

## Results and Discussion

### Experimental setup

#### Datasets

We use twelve datasets, each being a matrix in which a row corresponds to an element to be clustered and a column to an experimental condition. Since the aim of this study is to assess the performance of FC both with respect to Consensus and to other internal validation measures, a natural selection consists of the following two groups of datasets.

The first one, referred to as Benchmark 1, is composed of six datasets, each referred to as Leukemia, Lymphoma, CNS Rat, NCI60, PBM and Yeast. They have been widely used for the design and precision analysis of internal validation measures, e.g., [10,11,22,24,34], that are now mainstays of this discipline. Indeed, they seem to be a *de facto *standard, offering the advantage of making this work comparable with methods directly related to FC. In particular, by using them we are able to use the entire experimentation by Giancarlo et al. in order to assess the performance of FC relative to other validation measures. The second group, referred to as Benchmark 2, is composed of six datasets, taken from Monti et al., that nicely complement the datasets in Benchmark 1. Their selection allows for a direct comparison of the performance of FC with Consensus on datasets that were originally used for its validation. Each of those datasets is referred to as Normal, Novartis, St. Jude, Gaussian3, Gaussian5 and Simulated6, the last three being artificial data.

Since all of the mentioned datasets have been widely used in previous studies, we provide only a synoptic description of each of them in the Supplementary File, where the interested reader can find relevant references for a more in-depth description of them. However, it seems appropriate to recall some of their key features here. The datasets in Benchmark 1 have relatively few items to classify and relatively few dimensions (at most 200 hundred-see the Supplementary File). However, it is worth mentioning that Lymphoma, NCI60 and Leukemia have been obtained by Dudoit and Fridlyand and Handl et al., respectively, via an accurate statistical screening of the three relevant microarray experiments that involved thousands of conditions (columns). That screening process eliminated most of the conditions since there was no statistically significant variation across items (rows). It is also worth pointing out that the three mentioned datasets are quite representative of microarray cancer studies. The CNS Rat and Yeast datasets come from gene functionality studies. The fifth one, PBM, is a dataset that corresponds to a cDNA with a large number of items to classify and it is used to show the current limitations of existing validation methods that have been outlined in (B) in the Background section. Indeed, those limits have been established with PBM as input. In particular, when given to Consensus as input, the computational demand is such that all experiments were stopped after four days, or they would have taken weeks to complete.

Except for one, the datasets in Benchmark 2 are all of very high dimension (at most 1277-see the Supplementary File). The artificial ones were designed by Monti et al. to assess the ability of Consensus to deal with clustering scenarios typical of microarray data, as detailed in the Supplementary File. Therefore, in experimenting with them, we test whether key features of Consensus are preserved by FC. Moreover, the three microarrays are all cancer studies that preserve their high dimensionality even after statistical screening, as opposed to the analogous datasets in Benchmark 1.

We also mention that all datasets have a "gold solution", i.e., a partition of the data into a number of classes known *a priori *or that has been validated by experts. A technical definition of gold solution is reported in the Supplementary File. Here we limit ourselves to mention that we adhere to the methodology reported in Dudoit and Fridlyand.

#### Clustering algorithms and dissimilarity matrices

We use hierarchical, partitional clustering algorithms [13] and NMF when viewed as a clustering algorithm. In particular, the hierarchical methods used are Hier-A (Average Link), Hier-C (Complete Link) and Hier-S (Single Link). We use each of K-means and NMF, both of them in the version that starts the clustering from a random partition of the data, with acronyms K-means-R and NMF-R, and in the version where each takes, as part of its input, an initial partition produced by one of the chosen hierarchical methods. For K-means, the acronym for those latter versions are K-means-A, K-means-C and K-means-S, respectively. An analogous notation is followed for NMF. Following Giancarlo et al., all of our algorithms use Euclidean distance in order to assess the similarity of single elements to be clustered. The interested reader will find a detailed discussion about this choice in Giancarlo et al.. Since NMF is relatively novel in the biological data mining literature, it is described with considerable detail in the Supplementary File, for the convenience of the reader.

#### Hardware

All experiments for the assessment of the precision of each measure were performed in part on several state-of-the-art PCs and in part on a 64-bit AMD Athlon 2.2 GHz bi-processor with 1 GB of main memory running Windows Server 2003. All the timing experiments reported were performed on the bi-processor, using one processor per run. The use of several machines for the experimentation was deemed necessary in order to complete the full set of experiments in a reasonable amount of time. Indeed, as detailed later, some experiments would require weeks to complete execution on PBM, the largest dataset we have used. Indeed, we anticipate that some experiments were stopped after four days, because it was evident that they would have taken weeks to complete. We also point out that all the Operating Systems supervising the computations have a 32 bits precision.

### Consensus and its parameters

It is helpful for the discussion to highlight, here, some key facts about Consensus, deferring the detailed description of the procedure to the Methods section. For a given number of clusters, Consensus computes a certain number of clustering solutions (resampling step), each from a sample of the original data (subsampling). The performance of Consensus depends on two parameters: the number of resampling steps *H *and the percentage of subsampling *p*, where *p *states how large the sample must be. From each clustering solution, a corresponding connectivity matrix is computed: each entry in that matrix indicates whether a pair of elements is in the same cluster or not. For the given number of clusters, the consensus matrix is a normalized sum of the corresponding *H *connectivity matrices. Intuitively, the consensus matrix indicates the level of agreement of clustering solutions that have been obtained via independent sampling of the dataset.

Monti et al., in their seminal paper, set *H *= 500 and *p *= 80%, without any experimental or theoretical justification. For this reason and based also on an open problem mentioned in [20], we perform several experiments with different parameter settings of *H *and *p*, in order to find the "best" precision-time trade-off, when Consensus is regarded as an internal validation measure.

In order to assess a good parameter setting for Consensus, using the hierarchical algorithms and K-means, we have performed experiments with *H *= 500, 250, 100 and *p *= 80%, 66%, respectively, on the Benchmark 1 datasets, reporting the precision values and times. The choice of the value of *p *is justified by the results reported in [22,23]. Intuitively, a value of *p *smaller then 66% would fail to capture the entire cluster structure present in the data.

For each dataset and each clustering algorithm-except NMF (see below), we compute Consensus for a number of cluster values in the range [2,30] , while, for Leukemia, the range [2,25] is used when *p *= 66%, due to its small size. Therefore, for this particular dataset, the timing results are not reported since incomparable with the ones obtained with the other datasets. The prediction value, k*, is based on the plot of the Δ(*k*) curve, with the possible consideration also of the CDF curves, (both types of curves are defined in the Methods section) as indicated in [19,20]. The corresponding plots are available at the supplementary material web site, in the Figures section, where they are organized by benchmark dataset-internal validation measure-subsampling size-number of resampling steps. The corresponding tables summarizing the prediction and timing results are again reported at the supplementary material web site, in the Tables section, and they follow the same organization outlined for the Figures. For reasons that will be evident shortly and due to its high computational demand, we have performed experiments only with *H *= 250 and *p *= 80% in conjunction with NMF.

For *p *= 80%, the precision results reported in the corresponding tables at the supplementary material web site show that there is very little difference between the results obtained for *H *= 500 and *H *= 250. That is in contrast with the results for *H *= 100, where many prediction values are very far from the gold solution for the corresponding dataset, e.g., the Lymphoma dataset. Such a finding seems to indicate that, in order to find a consensus matrix which captures well the inherent structure of the dataset, one needs a sensible number of connectivity matrices. The results for a subsampling value of *p *= 66% confirms that the number of connectivity matrices one needs to compute is more relevant than the percentage of the data matrix actually used to compute them. Indeed, although it is obvious that a reduction in the number of resampling steps results in a saving in terms of execution time, it is less obvious that for subsampling values *p *= 66% and *p *= 80%, there is no substantial difference in the results, both in terms of precision and of time. Therefore, a proper parameter setting for Consensus seems to be *H *= 250 and *p *= 80%. In regard to NMF, we have used only that parameter setting for our experiments. For later use, we report in Table 1 part of the results of the experiments with the parameter setting *H *= 250 and *p *= 80%. Indeed, as for timing results, we report only the ones for CNS Rat, NCI60, PBM and Yeast datasets since the ones for Leukemia and Lymphoma are comparable to those obtained for CNS Rat and NCI60 and therefore are redundant. As for the Benchmark 2 datasets, we have experimented only with the parameter setting *H *= 250 and *p *= 80%. For each dataset and each algorithm, the predictions have been derived in analogy with the ones for datasets in Benchmark 1. The relevant figures and tables are at the supplementary material web site, again organized in analogy with the criteria described for Benchmark 1. For later use, we report the table here as Table 2. For the artificial datasets, we do not report the timing results since the experiments have been performed on a computer other than the AMD Athlon.

**Table 1:** Results for Consensus with H = 250 and p = 80% on the Benchmark 1 datasets

	Precision						Timing			
	**CNS Rat**	**Leukemia**	**NCI60**	**Lymph**.	**Yeast**	**PBM**	**CNS Rat**	**NCI60**	**Yeast**	**PBM**

Hier-A	⑦	➌	➑	➌	➎	-	8.9 × 10^5^	1.4 × 10^6^	5.0 × 10^7^	-
Hier-C	➏	④	➑	$\begin{array}{c} 5 \end{array}$	⑥	-	8.1 × 10^5^	1.3 × 10^6^	4.8 × 10^7^	-
Hier-S	2	➌	$\begin{array}{c} 10 \end{array}$	②	10	-	4.3 × 10^5^	1.0 × 10^5^	4.8 × 10^7^	-
K-means-R	➏	④	⑦	④	⑥	-	5.6 × 10^5^	1.2 × 10^6^	2.7 × 10^7^	-
K-means-A	⑦	➌	➑	➌	⑥	-	1.0 × 10^6^	1.8 × 10^6^	5.6 × 10^7^	-
K-means-C	➏	➌	➑	④	⑥	-	9.8 × 10^5^	1.7 × 10^6^	5.3 × 10^7^	-
K-means-S	⑦	$\begin{array}{c} 5 \end{array}$	⑨	②	⑥	-	1.2 × 10^6^	1.2 × 10^6^	5.7 × 10^7^	-
NMF-R	➏	④	⑦	④	-	-	1.1 × 10^8^	6.4 × 10^7^	-	-
NMF-A	⑦	➌	2	➌	-	-	3.0 × 10^7^	1.3 × 10^7^	-	-
NMF-C	5	④	⑦	④	-	-	3.0 × 10^7^	1.3 × 10^7^	-	-
NMF-S	2	8	⑨	②	-	-	3.6 × 10^7^	1.3 × 10^7^	-	-

**Gold solution**	**6**	**3**	**8**	**3**	**5**	**18**	-	-	-	-

A summary of the results for Consensus with *H *= 250 and *p *= 80%, on all algorithms, on the Benchmark 1 datasets. Each cell in the table displays either a precision or a timing result. That is, either the prediction of the number of clusters in a dataset given by a measure or the execution time it took to get such a prediction. For cells displaying precision, a number in a circle with a black background indicates a prediction in agreement with the number of classes in the dataset; while a number in a circle with a white background indicates a prediction that differs, in absolute value, by 1 from the number of classes in the dataset; a number in a square indicates a prediction that differs, in absolute value, by 2 from the number of classes in the dataset; a number not in a circle/square indicates the remaining predictions. When one obtains two very close predictions for k*, they are both reported and separated by a dash. An entry containing a dash only indicates that either the experiment was stopped because of its high computational demand or that no useful indication was given by the method. For cells displaying timing, we use the following notation. Numeric values report timing in milliseconds, while a dash indicates that the timing is not available for at least one of the following reasons: the experiment (a) was performed on a computer other than the AMD Athlon; (b) it was stopped because of its high computational demand; (c) a smaller range of clustering solutions have been produced for that dataset, due to its size, i.e., Leukemia with *p *= 66%. For this particular set of experiments, we do not report the timing results for Leukemia and Lymphoma because they are redundant.

**Table 2:** Results for Consensus with H = 250 and p = 80% on the Benchmark 2 datasets

	Precision						Timing		
	**Novartis**	**St.Jude**	**Normal**	**Gaussian3**	**Gaussian5**	**Simulated6**	**Novartis**	**St.Jude**	**Normal**

Hier-A	⑤ - $\begin{array}{c} 6 \end{array}$	➏	10	➌	➎	⑤	1.0 × 10^7^	3.7 × 10^7^	9.5 × 10^6^
Hier-C	➍- ⑤	⑤ - ➏	10	➌	➎	⑤	1.0 × 10^7^	3.7 × 10^7^	9.2 × 10^6^
Hier-S	⑤	2	10	②	2	⑦	9.8 × 10^6^	3.7 × 10^7^	9.4 × 10^6^
K-means-R	⑤	➏	10	➌	➎	➏	1.8 × 10^7^	1.5 × 10^7^	6.3 × 10^6^
K-means-A	⑤ - $\begin{array}{c} 6 \end{array}$	➏	8	➌	➎	⑤	1.4 × 10^7^	6.8 × 10^7^	1.1 × 10^7^
K-means-C	➍- ⑤	⑤ - ➏	10	➌	➎	⑤	1.5 × 10^7^	6.8 × 10^7^	1.0 × 10^7^
K-means-S	⑤	➏	10	②	➎	➏	1.6 × 10^7^	6.8 × 10^7^	1.1 × 10^7^

**Gold solution**	**4**	**6**	**13**	**3**	**5**	**6**	-	-	-

A summary of the results for Consensus with *H *= 250 and *p *= 80%, on all algorithms, except NMF, and for the datasets in Benchmark 2. The table legend is as in Table 1. NMF has been excluded since each experiment was terminated due to its high computational demand. The timing results for the artificial datasets are not reported since the experiments have been performed on a computer other than the AMD Athlon.

From our experiments, in particular the ones on the Benchmark 1 datasets, several conclusons can be draw. A simple reduction in terms of *H *and *p *is not enough to grant a good precision-time trade-off. Even worse, although the parameter setting *H *= 250 and *p *= 80% grants a faster execution of Consensus with respect to the original setting by Monti et al., the experiments on the PBM dataset were stopped after four days on all algorithms. That is, the largest of the datasets used here is still "out of reach" of Consensus even with a tuning of the parameters aimed at reducing its computational demand. Such a finding, together with the state of the art outlined in the Background section, motivates our interest in the design of alternative methods, such as fast heuristics.

Regarding NMF, its inefficiencies compound with those of Consensus; that is, the relatively large number of connectivity matrices needed by Consensus and the well-known slow convergence of NMF for the computation of a clustering solution, since connectivity matrices are obtained from clustering solutions. The end-result is a slow-down of one order of magnitude with respect to Consensus used in conjunction with other clustering algorithms. As a consequence, NMF and Consensus can be used together on a conventional PC only for relatively small datasets. In fact, the experiments for Yeast and PBM, the two largest datasets in Benchmark 1 with which we have experimented, were stopped after four days. An analogous outcome was observed for the experiments on all of the microarray datasets in Benchmark 2.

### FC and its parameters

In analogy with Consensus, the precision and time performances of FC depend on *H *and *p*. In order to validate this measure, we repeat verbatim the experiments that have been performed here for Consensus. The relevant information is in the Figures and Tables section of the supplementary material web site and it follows the same organization as the one described for Consensus. Again, we find that the "best" parameter setting is *H *= 250 and *p *= 80% also for FC. The tables of interest are reported here as Table 3 and 4 and they are used for the comparison with Consensus.

**Table 3:** Results for FC with H = 250 and p = 80% on the Benchmark 1 datasets

	Precision						Timing			
	**CNS Rat**	**Leukemia**	**NCI60**	**Lymph**.	**Yeast**	**PBM**	**CNS Rat**	**NCI60**	**Yeast**	**PBM**

Hier-A	⑦	➌	➑	➌	➎	2	4.7 × 10^4^	5.2 × 10^4^	1.4 × 10^6^	3.7 × 10^7^
Hier-C	➏	④	➑	$\begin{array}{c} 5 \end{array}$	⑥	14 - ⑰	4.4 × 10^4^	6.4 × 10^4^	1.4 × 10^6^	3.7 × 10^7^
Hier-S	2	8	➑	②	10	2	5.3 × 10^4^	5.2 × 10^4^	1.4 × 10^6^	3.0 × 10^7^
K-means-R	➏	④	⑦	④	⑥	$\begin{array}{c} 16 \end{array}$	3.7 × 10^5^	1.2 × 10^6^	1.6 × 10^7^	1.6 × 10^8^
K-means-A	⑦	➌	➑	➌	⑥	12	3.1 × 10^5^	9.3 × 10^5^	1.8 × 10^7^	2.1 × 10^8^
K-means-C	➏	④	➑	④	⑥	12	2.5 × 10^5^	6.5 × 10^5^	1.4 × 10^7^	2.0 × 10^8^
K-means-S	➏	7	⑨	②	⑥	2	3.7 × 10^5^	6.9 × 10^5^	1.9 × 10^7^	2.4 × 10^8^
NMF-R	➏	④	⑦	④	-	-	1.1 × 10^8^	6.3 × 10^7^	-	-
NMF-A	⑦	➌	⑦	➌	-	-	3.0 × 10^7^	1.2 × 10^7^	-	-
NMF-C	➏	➌	➑	④	-	-	2.9 × 10^7^	1.2 × 10^7^	-	-
NMF-S	2	8	⑨	②	-	-	3.5 × 10^7^	1.2 × 10^7^	-	-

**Gold solution**	**6**	**3**	**8**	**3**	**5**	**18**	-	-	-	-

A summary of the results for FC with *H *= 250 and *p *= 80%, on all algorithms and on the Benchmark 1

datasets. The table legend is as in Table 1.

**Table 4:** Results for FC with H = 250 and p = 80% on the Benchmark 2 datasets

	Precision						Timing		
	**Novartis**	**St.Jude**	**Normal**	**Gaussian3**	**Gaussian5**	**Simulated6**	**Novartis**	**St.Jude**	**Normal**

Hier-A	⑤ - $\begin{array}{c} 6 \end{array}$	➏	10	➌	➎	⑤	4.0 × 10^5^	1.6 × 10^6^	3.4 × 10^5^
Hier-C	➍ - ⑤	⑤ - ➏	10	➌	➎	⑤	3.9 × 10^5^	1.4 × 10^6^	3.3 × 10^5^
Hier-S	⑤	2	10	②	2	⑦	4.4 × 10^5^	1.5 × 10^6^	3.4 × 10^5^
K-means-R	⑤	➏	10	➌	➎	➏	1.4 × 10^7^	5.9 × 10^6^	2.0 × 10^6^
K-means-A	⑤ - $\begin{array}{c} 6 \end{array}$	➏	8	➌	➎	⑤	5.5 × 10^6^	3.2 × 10^7^	5.4 × 10^6^
K-means-C	➍ - ⑤	⑤ - ➏	10	➌	➎	⑤	6.5 × 10^6^	3.2 × 10^7^	2.1 × 10^6^
K-means-S	⑤	➏	10	②	➎	➏	7.8 × 10^6^	4.9 × 10^7^	2.1 × 10^6^

**Gold solution**	**4**	**6**	**13**	**3**	**5**	**6**	-	-	-

A summary of the results for FC with *H *= 250 and *p *= 80%, on all algorithms, except NMF, and for the datasets in Benchmark 2. The table legend is as in Table 1. NMF has been excluded since each experiment was terminated due to its high computational demand. The timing results for the artificial datasets are not reported since the experiments have been performed on a computer other than the AMD Athlon.

Consider Table 1 and 3. Note that, in terms of precision, FC and Consensus provide identical predictions on the Lymphoma and Yeast datasets, while their predictions are quite close on the CNS Rat dataset. Moreover, in terms of time, note that FC is faster then Consensus by at least one order of magnitude on all hierarchical algorithms and K-means-A, K-means-C and K-means-S. In particular, FC is able to complete execution on the PBM dataset, as opposed to Consensus, with all of the mentioned algorithms. It is also worthy of notice that Hier-C and K-means-R also provide, for the PBM dataset, a reasonable estimate of the number of clusters present in it. Finally, the one order of magnitude speed-up is preserved with increasing values of *H*. That is, as *H *increases the precision of both Consensus and FC increases, but the speed-up of FC with respect to Consensus is preserved (see the timing results reported at the supplementary material web site for *H *= 500, 250, 100 and *p *= 80% on the Benchmark 1 datasets). It is somewhat unfortunate, however, that those quite substantial speed-ups have only minor effects when one uses NMF as a clustering algorithm, which is a clear indication that the time taken by NMF to converge to a clustering solution accounts for most of the time performance of FC in that setting, in analogy with Consensus.

Consider now Table 2 and 4. Based on them, it is of great interest to notice that, on the datasets of Benchmark 2, there is no difference whatsoever in the predictions between Consensus and FC. Even more remarkably, by analyzing the Δ and the *CDF *curves from which the predictions are made (see Methods section), one discovers that the ones produced by Consensus and FC are nearly identical (see Figs. M1-M24 at the supplementary material web site). However, on the microarray datasets in Benchmark 2, FC is at least one order of magnitude faster than Consensus, with exactly the same algorithms indicated for Benchmark 1. NMF results to be problematic also on the Benchmark 2 datasets.

### Comparison of FC with other internal validation measures

It is also of interest to compare FC with other validation measures that are available in the literature. We take, as reference, the benchmarking results reported in Giancarlo et al., since for the Benchmark 1 datasets the experimental setup is identical to the one used here. As mentioned in the Background section, that benchmarking accounts for the three most widely known families of validation measures: namely, those based on (a) hypothesis testing in statistics; (b) stability-based techniques and (c) jackknife techniques, in particular, the Gap Statistics for category (a); CLEST [22], Model Explorer [23] and Consensus for category (b) and FOM for category (c). Moreover, there are also included G-Gap, an approximation of the Gap Statistics, and one extension of FOM, referred to as Diff-FOM. In addition, that study takes into account two classical measures as WCSS and the KL (Krzanowski and Lai index) [35]. In the Supplementary File, a short description is given of the measures relevant to this discussion.

Using the Benchmark 1 datasets, Giancarlo et al. show there is a natural hierarchy, in terms of time, for those measures. Moreover, the faster the measure, the less accurate it is. From that study and for completeness, we report in Table TI13, at the supplementary material web site, the best performing measures, with the addition of FC. From that table, we extract and report, in Table 5 the fastest and best performing measures - again, with the addition of FC. Consistent with that study, we report the timing results only for CNS Rat, Leukemia, NCI60 and Lymphoma. As is self-evident from that latter table, FC with Hier-A is within a one order of magnitude difference in speed with respect to the fastest measures, i.e., WCSS and G-Gap. Quite remarkably, it grants a better precision in terms of its ability to identify the underlying structure in each of the benchmark datasets. It is also of relevance to point out that FC with Hier-A has a time performance comparable to that of FOM, but again it has a better precision performance. Notice that, none of the three just-mentioned measures depends on any parameter setting, implying that no speed-up will result from a tuning of the algorithms.

**Table 5:** Summary of results for the fastest measures on the Benchmark 1 datasets

	Precision					Timing			
	**CNS Rat**	**Leukemia**	**NCI60**	**Lymphoma**	**Yeast**	**CNS Rat**	**Leukemia**	**NCI60**	**Lymphoma**

WCSS-K-means-C	⑤	➌	➑	8	④	1.7 × 10^3^	1.3 × 10^3^	5.0 × 10^3^	4.0 × 10^3^
WCSS-R-R0	⑤	④	➑	➌	④	1.2 × 10^3^	8.0 × 10^2^	4.1 × 10^3^	3.0 × 10^3^
G-Gap-K-means-R	⑦	➌	4	④	⑥	2.4 × 10^3^	2.0 × 10^3^	8.3 × 10^4^	8.4 × 10^3^
G-Gap-R-R5	⑤	④	2	②	④	1.2 × 10^3^	8.0 × 10^2^	4.5 × 10^4^	3.2 × 10^3^
FOM-K-means-C	⑦	8	➑	④	④	1.9 × 10^4^	9.4 × 10^4^	5.5 × 10^5^	2.6 × 10^5^
FOM-K-means-S	➏	➌	➑	8	④	2.9 × 10^4^	1.0 × 10^5^	7.1 × 10^5^	3.6 × 10^5^
FOM-R-R5	➏	➌	⑦	$\begin{array}{c} 5 \end{array}$	➎	3.9 × 10^3^	3.7 × 10^4^	2.1 × 10^5^	7.6 × 10^4^
FOM-Hier-A	⑦	➌	⑦	6	⑥	1.6 × 10^3^	7.5 × 10^3^	5.1 × 10^4^	1.8 × 10^4^
DIFF-FOM-K-means-C	⑦	➌	⑦	④	$\begin{array}{c} 3 \end{array}$	1.9 × 10^4^	9.4 × 10^4^	5.5 × 10^5^	2.6 × 10^5^
FC-Hier-A	⑦	➌	➑	➌	➎	5.9 × 10^4^	2.7 × 10^4^	7.0 × 10^4^	6.8 × 10^4^
FC-Hier-C	➏	④	➑	$\begin{array}{c} 5 \end{array}$	⑥	5.9 × 10^4^	2.7 × 10^4^	6.5 × 10^4^	6.7 × 10^4^

**Gold solution**	**6**	**3**	**8**	**3**	**5**	-	-	-	-

A summary of the best performing measures taken from the benchmarking of Giancarlo et al., with the addition of FC, with *H *= 250 and *p *= 80%. The table legend is as in Table 1. Consistent with that study, we report only the timing results for CNS Rat, Leukemia, NCI60 and Lymphoma, since for the Yeast and PBM datasets the experiments have been performed on a computer other than the AMD Athlon.

For completeness and in order to even better assess the precision merits of FC with respect to the measures considered in Table TI13, we have performed experiments also on the Benchmark 2 datasets. The timing results are not reported since the experiments have been performed on computers other than the AMD Athlon. Since most of the methods in that table predict k* based on the identification of a "knee" in a curve (in analogy with the Δ curve of Consensus), the relevant figures are reported at the supplementary material web site. Table TI16, at the supplementary material web site, summarizes the results. We extract from Table TI16 the same measures present in Table 5 and report them in Table 6. Again, FC is among the most precise.

**Table 6:** Summary of results for the fastest measures on the Benchmark 2 datasets

	Precision					
	**Novartis**	**St. Jude**	**Normal**	**Gaussian3**	**Gaussian5**	**Simulated6**

WCSS-K-means-C	⑤	➏	9	➌	$\begin{array}{c} 4 \end{array}$	⑦
WCSS-R-R0	⑤	➏	9	-	➌	⑦
G-Gap-K-means-R	⑦	$\begin{array}{c} 4 \end{array}$	7	➌	8	3
G-Gap-R-R5	⑤	⑦	7	➌	$\begin{array}{c} 7 \end{array}$	3
FOM-K-means-C	➍	⑤	6	➌	-	⑤
FOM-K-means-S	➍	⑦	4	-	-	$\begin{array}{c} 4 \end{array}$
FOM-R-R5	7	-	10	➌	-	⑦
FOM-Hier-A	➍	8	6	➌	-	⑤
DIFF-FOM-K-means-C	⑦	3	7	➌	29	3
FC-Hier-A	5 - $\begin{array}{c} 6 \end{array}$	➏	10	➌	➎	⑤
FC-Hier-C	➍ - ⑤	⑤ - ➏	10	➌	➎	⑤

**Gold solution**	**4**	**6**	**13**	**3**	**5**	**6**

A summary of the best performing measures taken from the benchmarking of Giancarlo et al., with the addition of FC, with *H *= 250 and *p *= 80%. The table legend is as in Table 1. The timing results are not reported since the experiments have been performed on a computer other than the AMD Athlon.

The results outlined above are particularly significant since (i) FOM is one of the most established and highly-referenced measures specifically designed for microarray data; (ii) in purely algorithmic terms, WCSS and G-Gap, are so simple as to represent a "lower bound" in terms of the time performance that is achievable by any data-driven internal validation measure. In conclusion, our experiments show that FC is quite close in time performance to three of the fastest data-driven validation measures available in the literature, while also granting better precision results. In view of the fact that the former measures are considered reference points in this area, the speed-up of Consensus proposed here seems to be a non-trivial step forward in the area of data-driven internal validation measures.

## Conclusions

FC is an algorithm that guarantees a speed-up of at least one order of magnitude with respect to Consensus, when used in conjunction with hierarchical clustering algorithms or with partitional algorithms with a hierarchical initialization. Remarkably, it preserves what seem to be the most outstanding properties of that measure: the accuracy in identifying structure in the input dataset and the ability to produce a dissimilarity matrix that can be used to improve the performance of clustering algorithms. For this latter point-see the Supplementary File. Moreover, the speed-up does not seem to depend on the number *H *of resampling steps.

In terms of the existing literature on data-driven internal validation measures, we have that, by extending the benchmarking results of Giancarlo et al., FC is only one order of magnitude away from the fastest measures, i.e., WCSS, yet granting a superior performance in terms of precision. Although FC does not close the gap between the time performance of the fastest internal validation measures and the most precise, it is a substantial step forward towards that goal. For one thing, its time performance is comparable with that of FOM and with a better precision, a result of great significance in itself, given the fact that FOM is one of the oldest and most prestigious methods in the microarray data analysis area.

Furthermore, some conclusions that are of interest from the methodological point of view can also be drawn. The idea of "approximating" an internal validation measure to achieve a speed-up, introduced by Giancarlo et al. and applied here to Consensus, seems to lead to significant improvements in time performance with minor losses in predictive power. As detailed in the Methods section, the technique we have developed here, although admittedly simple, can be used in other contexts, where a given number of clustering solutions must be computed from different samples of the same dataset. That is a typical scenario common to many stability-based validation measures, i.e., [22,23,28,29,31,36].

Unfortunately, FC, when used in conjunction with NMF, is almost as slow as Consensus. Those experiments provide additional methodological, as well as pragmatic, insights affecting both clustering and pattern discovery in biological data. Indeed, although the work by Brunet et al. uses Consensus in conjunction with NMF in order to identify the number of clusters in a dataset, our experiments show that both Consensus and FC have a better prediction power when used with Hier-A and Hier-C than when used with NMF. In view of the steep computational price one must pay, the use of NMF with Consensus and FC does not seem to be justified. Indeed, the major contribution given by Brunet et al. in their seminal paper is to show that NMF can give a succinct representation of the data, which can then be used for pattern discovery. Our work shows that, as far as clustering and validation measures go, choices more convenient than NMF are possible: it is somewhat reductive to consider NMF as a clustering algorithm.

In summary, FC with a parameter setting, i.e, *H *= 250 and *p *= 80%, that makes it robust with respect to small and medium-sized datasets, i.e, number of items to cluster in the hundreds and number of conditions up to a thousand, seems to be the internal validation measure of choice. It remains open to establish a good parameter setting for datasets with thousands of elements to cluster. Given the current state of the art, addressing such a question means to come-up with an internal validation measure able to correctly predict structure when there are thousands of elements to classify. A task far from obvious, given that all measures in the benchmarking by Giancarlo et al. have serious limitation in their predictive power for datasets with a number of elements in the thousands.

## Methods

### Consensus

Consensus is a stability-based technique, which is best presented as a procedure taking as input *Sub*, *H*, *D*, *A*, *k_max_*. The resampling scheme *Sub *is a means of sampling from one dataset in order to build a new one. In our experiments, the resampling scheme extracts, uniformly and at random, a given percentage *p *of the rows of the dataset *D*. Finally, *H *is the number of resampling steps, *A *is the clustering algorithm and *k_max _*is the maximum number that is considered as candidate for the "correct" number k* of clusters in *D*.

Procedure Consensus(*Sub*, *H*, *D*, *A*, *k*_*max*_)

**(1) **For 2 ≤ *k *≤ *k_max_*, initialize to empty the set *M *of connectivity matrices and perform steps **(1.a) **and **(1.b)**.

**(1.a) **For 1 ≤ *h *≤ *H*, compute a perturbed data matrix *D*^(*h*) ^using resampling scheme *Sub*; cluster the elements in *k *clusters using algorithm *A *and *D*^(*h*)^. Compute a connectivity matrix *M*^(*h*) ^and insert it into *M*.

**(1.b) **Based on the connectivity matrices in *M*, compute a consensus matrix ${ℳ}^{\left(k\right)}$.

**(2) **Based on the *k_max _*- 1 consensus matrices, return a prediction for k*.

As for the connectivity matrix *M*^(*h*)^, one has *M*^(*h*)^(*i*, *j*) = 1 if items *i *and *j *are in the same cluster and zero otherwise. Moreover, we also need to define an indicator matrix *I*^(*h*) ^such that *I*^(*h*)^(*i*, *j*) = 1 if items *i *and *j *are both in *D*^(*h*) ^and zero otherwise. Then, the consensus matrix ${ℳ}^{\left(k\right)}$ is defined as a properly normalized sum of all connectivity matrices in all perturbed datasets:

(1) $${ℳ}^{\left(k\right)}=\frac{\sum_{h}M^{\left(h\right)}}{\sum_{h}I^{\left(h\right)}}$$

**Figure 1:**
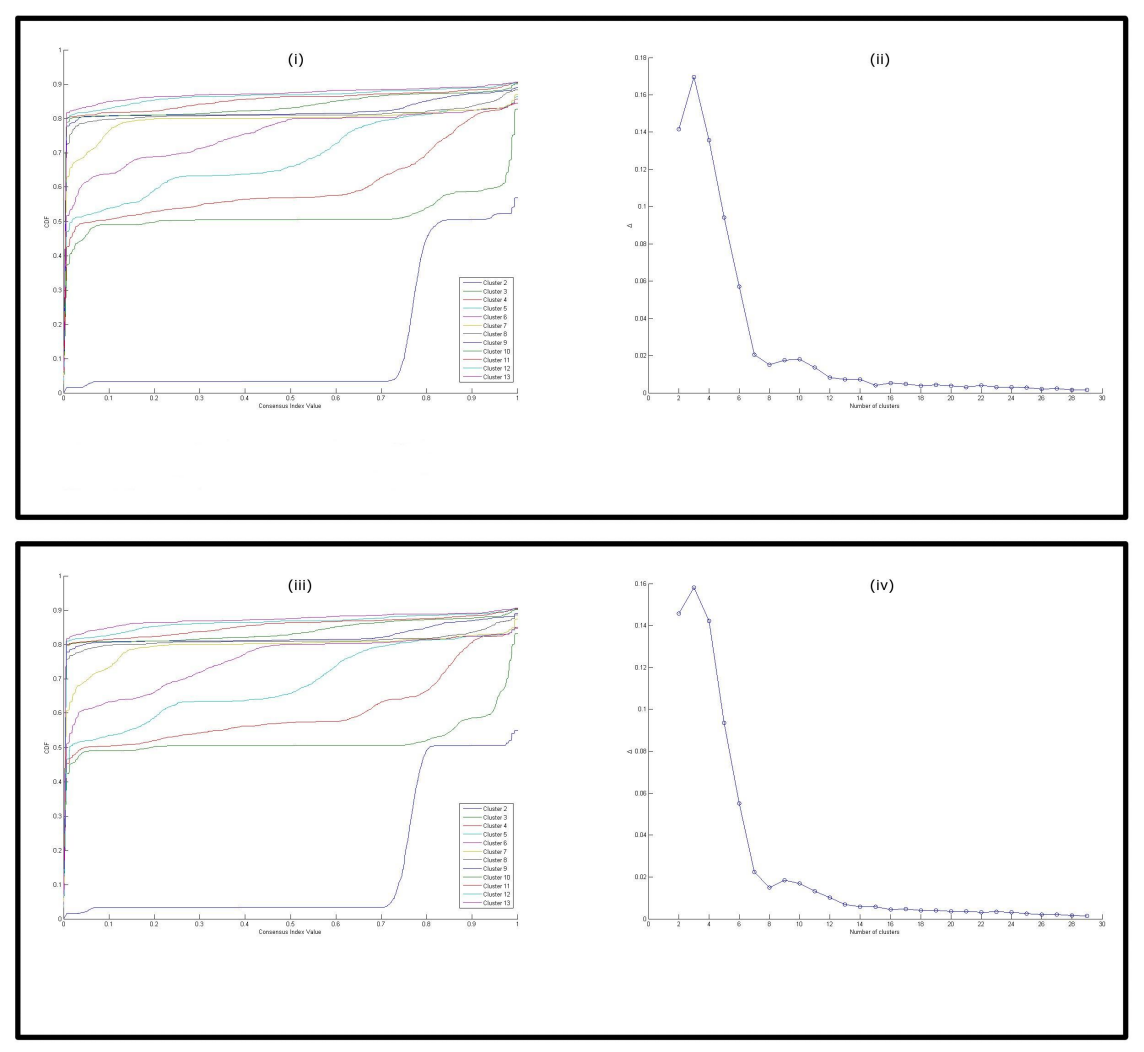
**An example of number of cluster prediction with the use of Consensus and FC**. The experiment is derived with the Leukemia dataset as input, with the use of the K-means-A clustering algorithm. (i) The plot of the CDF curves as a function of *k*, obtained by Consensus with *H *= 250 and *p *= 80%. For clarity, only the curves for *k *in [2, 13] are shown. It is evident that there are increasing values of the area under the *CDF *for increasing values of *k*. The flattening effect in the growth rate of the area is evident for *k *≥ k* = 3. (ii) The plot of the corresponding Δ curve for *k *in [2, 30], where the flattening effect indicating k* is evident for *k *≥ k* = 3. (iii) The plot of the *CDF *curves, obtained by FC with *H *= 250 and *p *= 80%, in analogy with (i). (iv) The plot of the Δ curve, obtained by FC with *H *= 250 and *p *= 80%, in analogy with (ii).

Based on experimental observations and sound arguments, Monti et al. derive a "rule of thumb" in order to estimate the real number k* of clusters present in *D*. Here we limit ourselves to present the key points, since the interested reader can find a full discussion in Monti et al.. Let *n *be the number of items to cluster, *m *= *n*(*n *- 1)/2, and {*x*_1_, *x*_2_, ⋯, *x_m_*} be the list obtained by sorting the entries of the consensus matrix. Moreover, let the empirical cumulative distribution *CDF*, defined over the range [0, 1], be:

$$CDF(c)=\frac{\sum_{i<j}l\{ℳ(i,j)\;\;\leq \;\;c\}}{m}$$

where *c *is a chosen constant in [0, 1] and *l *equals one if the condition is true and it is zero otherwise. For a given value of *k*, i.e., number of clusters, consider the *CDF *curve obtained by plotting the values of *CDF*(*x_i_*), 1 ≤ *i *≤ *m*, with the use of the corresponding consensus matrix. In an ideal situation in which there are *k *clusters and the clustering algorithm is so good to provide a perfect classification, such a curve is bimodal, with peaks at zero and one. Monti et al. observe and validate experimentally that the area under the *CDF *curves is an increasing function of *k*. That result has also been confirmed by the experiments in Giancarlo et al.. In particular, for values of *k *≤ k*, that area has a significant increase, while its growth flattens out for *k *>k*. For instance, with reference to Figure 1 one sees an increase in the area under the *CDF*s for *k *= 2, ..., 13. The growth rate of the area is decreasing as a function of *k *and it flattens out for *k *≤ k* = 3. The point in which such a growth flattens out can be taken as an indication of k*. However, operationally, Monti et al. propose a closely associated method, described next. For a given *k*, the area of the corresponding *CDF *curve is estimated as follows:

$$A\left(k\right)=\sum_{i=2}^{m}\left[x_{i}-x_{i-1}\right]\;CDF\left(x_{i}\right)$$

Again, *A*(*k*) is observed to be an increasing function of *k*, with the same growth rate as the *CDF *curves. Now, let

$$\Delta (k)=\{\begin{array}{ll} A(k) & k=2,\\ \frac{A(k+1)-A(k)}{A(k)} & k>2. \end{array}$$

be the proportion increase of the *CDF *area as a function of *k *and as estimated by *A*(*k*). Again, Monti et al. observe experimentally that:

(i) For each *k *≤ k*, there is a pronounced decrease of the Δ curve. That is, the incremental growth of *A*(*k*) decreases sharply.

(ii) For *k *>k*, there is a stable plot of the Δ curve. That is, for *k *>k*, the growth of *A*(*k*) flattens out.

From this behavior, the "rule of thumb" to identify k* with the use of the Δ curve is: take as k* the abscissa corresponding to the smallest non-negative value where the curve starts to stabilize; that is, no big variation in the curve takes place from that point on. An example is given in Figure 1.

A few remarks are in order. From the observations outlines above, one has that, the value of the area under the *CDF *is not very important. Rather, its growth as a function of *k *is key. Moreover, experimentally, the Δ curve is non-negative. Such an observation has been confirmed by Giancarlo et al. However, there is no theoretic justification for such a fact. Even more importantly, the growth of the *CDF *curves also gives an indication of the number of clusters present in *D*. Such a fact, together with the use of the Δ curve, contributes to the quality of the prediction since *A*(*k*) is only an approximation of the real area under the *CDF *curve and it may give spurious indications that can be "disambiguated" with the use of the *CDF *curves. It is quite remarkable that there is usually excellent agreement in the prediction between the Δ curve and the *CDF *curves. For convenience of the reader, we recall here that many internal validation methods are based on the identification of a "knee" in a suitably defined curve, e.g., WCSS and FOM, in most cases via a visual inspection of the curve. For specific measures, there exist automatic methods that identify such a point, some of them being theoretically sound [21], while others are based on heuristic geometric observations [20,37]. For Consensus, the identification of a theoretically sound automatic method for the prediction of k* is open and it is not clear that heuristic approaches will yield appreciable results.

As stated in other parts of this paper, Consensus is quite representative of the area of internal validation measures. Indeed, the main, and rather simple, idea sustaining that procedure is the following. For each value of *k *in the range [2, *k_max_*], the procedure extracts *H *new data matrices from the original one and, for each of them, a partition into *k *clusters is generated. The better the agreement among those solutions, the higher the "evidence" that the value of *k *under scrutiny is a good estimate of k*. That level of agreement is measured via the consensus matrices. As clearly indicated in Handl et al., such a scheme is characteristic of stability-based internal validation measures. To the best our knowledge, the following methods are all the ones that fall in that class [22,23,28,29,31,36]. The main difference among them is how to predict k* once the *k_max _*× *H *clustering solutions have been generated, with a scheme that is the exact replica of the one adopted in Consensus. It is also worth noticing that each of the *k_max _*× *H *clustering solutions needed is computed from a distinct dataset. As is shown in the next section, this leads to inefficiencies, in particular in regard to agglomerative clustering algorithms, such as the hierarchical ones. Indeed, their ability to quickly compute a clustering solution with *k *clusters from one with *k *+ 1, typical of these methods, cannot be used within Consensus because, for each *k*, the dataset changes. The same holds true for divisive methods.

### FC

Intuitively, a large number of clustering solutions, each obtained via a sample of the original dataset, seem to be required in order to identify the correct number of clusters. However, there is no theoretic reason indicating that those clustering solutions must each be generated from a *different *sample of the input dataset, as Consensus does. Based on this observation, we propose to perform, first, a sampling step to generate a data matrix *D*^(*h*)^, which is then used to generate all clustering solutions for *k *in the range [2, *k_max_*]. In terms of code, that implies a simple switch of the two iteration cycles in steps (1) and (1.a) of the Consensus procedure. In turn, and with reference to the discussion at the end of the subsection regarding Consensus, that switch allows us to obtain a speed-up since costly computational duplications are avoided when the clustering algorithm *A *is hierarchical. Indeed, once the switch is done, it becomes possible to interleave the computation of the measure with the level bottom-up construction of the hierarchical tree underlying the clustering algorithms. Specifically, only one dendogram construction is required rather than the repeated and partial construction of dendograms as in the Consensus procedure. Therefore, we use, in full, the main characteristic of agglomerative algorithms briefly discussed in the subsection regarding Consensus. FC is formalized by the following procedure:

Procedure FC(*Sub, H, D, A, k*_*max*_)

**(1) **For 1 ≤ *h *≤ *H*, compute a perturbed data matrix *D*^(*h*) ^using resampling scheme *Sub*;.

**(1.a) **For 2 ≤ *k *≤ *k_max_*, initialize to empty the set *M*^(*k*) ^of connectivity matrices and cluster the elements in *k *clusters using algorithm *A *and *D*^(*h*)^. Compute a connectivity matrix *M*^(*h, k*)^.

**(2) **For 2 ≤ *k *≤ *k_max_*, based on the connectivity matrices in *M*, compute a consensus matrix ${ℳ}^{\left(k\right)}$.

**(3) **Based on the *k_max _- *1 consensus matrices, return a prediction for k*.

The "rule of thumb" one uses to predict k*, via FC, is the same as for Consensus. An example is reported in Figure 1(iii)-(iv). It is worth pointing out that both the CDFs and Δ curve shapes for FC closely track those of the respective curves for Consensus.

Finally, although the idea behind FC is simple, it has a general applicability that goes beyond the speed-up of Consensus. Indeed, as discussed earlier, all other stability-based methods available in the literature follow the same strategy for the construction of a large number of clustering solutions. Therefore, the FC speed-up applies also to them, although we have experimented only with Consensus since it seems to be the best, in terms of precision, in this category of measures.

## Availability

All software and datasets involved in our experimentation are available at the supplementary material web site. The software is given in a jar executable file for a Java Run Time environment. It works for Linux (various versions--see supplementary material web site), Mac OS X and Windows operating systems. Minimal system requirements are specified at the supplementary material web site, together with instructions and the binaries of K-means, hierarchical methods and NMF are also provided.

## Competing interests

The authors declare that they have no competing interests.

## Authors' contributions

RG and FU contributed equally to the design of the new algorithm, to the experimental methodology and to the write-up of the manuscript. FU implemented the algorithm and performed the experiments. RG directed the research. Both authors have read and approved the manuscript. This research by FU was conducted while in the Dipartimento di Matematica ed Informatica, Universitá di Palermo, Italy.

## References

[B1] Speed TP Statistical analysis of gene expression microarray data 2003 Chapman & Hall/CRC

[B2] Everitt B Cluster Analysis 1993 London: Edward Arnold

[B3] Hansen P Jaumard P Cluster Analysis and Mathematical Programming Mathematical Programming 1997 79 191 215

[B4] Hartigan JA Clustering Algorithms 1975 John Wiley and Sons

[B5] Jain AK Murty MN Flynn PJ Data Clustering: a Review ACM Computing Surveys 1999 31 3 264 323 10.1145/331499.331504

[B6] Kaufman L Rousseeuw PJ Finding Groups in Data: An Introduction to Cluster Analysis 1990 New York: Wiley

[B7] Mirkin B Mathematical Classification and Clustering 1996 Kluwer Academic Publisher

[B8] Rice JA Mathematical Statistics and Data Analysis 1996 Wadsworth

[B9] Hastie T Tibshirani R Friedman J The Elements of Statistical Learning 2003 Springer

[B10] Handl J Knowles J Kell DB Computational Cluster Validation in Post-genomic Data Analysis Bioinformatics 2005 21 15 3201 3212 10.1093/bioinformatics/bti517 15914541

[B11] Shamir R Sharan R Jiang T, Smith T, Xu Y, Zhang MQ Algorithmic Approaches to Clustering Gene Expression Data Current Topics in Computational Biology 2003 Cambridge, Ma.: MIT Press 120 161

[B12] D'haeseleer P How Does Gene Expression Cluster Work? Nature Biotechnology 2006 23 1499 1501 10.1038/nbt1205-1499 16333293

[B13] Jain A Dubes R Algorithms for Clustering Data 1988 Englewood Cliffs: Prentice-Hall

[B14] Seal S Comarina S Aluru S An Optimal Hierarchical Clustering Algorithm For Gene Expression Data Information Processing Letters 2004 93 143 147 10.1016/j.ipl.2004.11.001

[B15] Borodin A Ostrovsky R Rabani Y Subquadratic Approximation Algorithms for Clustering Problems in High Dimensional Space Machine Learning 2004 56 153 167 10.1023/B:MACH.0000033118.09057.80

[B16] Frahling G Sohler C A fast K-means Implementation Using Coresets Proceedings of the Twenty-Second Annual Symposium on Computational Geometry 2006 New York, NY, USA: ACM 135 143 full_text

[B17] Kraus J Kestler H A highly efficient multi-core algorithm for clustering extremely large datasets BMC Bioinformatics 2010 11 10.1186/1471-2105-11-169 20370922 PMC2865495

[B18] Milligan G Cooper M An Examination of Procedures for Determining the Number of Clusters in a Data Set Psychometrika 1985 50 159 179 10.1007/BF02294245

[B19] Monti S Tamayo P Mesirov J Golub T Consensus Clustering: A resampling-based Method for Class Discovery and Visualization of Gene Expression Microarray Data Machine Learning 2003 52 91 118 10.1023/A:1023949509487

[B20] Giancarlo R Scaturro D Utro F Computational cluster validation for microarray data analysis: experimental assessment of Clest, Consensus Clustering, Figure of Merit, Gap Statistics and Model Explorer BMC Bioinformatics 2008 9 462 10.1186/1471-2105-9-462 18959783 PMC2657801

[B21] Tibshirani R Walther G Hastie T Estimating the Number of Clusters in a Dataset via the Gap Statistics Journal Royal Statistical Society B 2001 2 411 423 10.1111/1467-9868.00293

[B22] Dudoit S Fridlyand J A Prediction-based Resampling Method for Estimating the Number of Clusters in a Dataset Genome Biology 2002 3 10.1186/gb-2002-3-7-research0036 12184810 PMC126241

[B23] Ben-Hur A Elisseeff A Guyon I A Stability Based Method for Discovering Structure in Clustering Data Seventh Pacific Symposium on Biocomputing 2002 ISCB 6 17 11928511

[B24] Yeung K Haynor D Ruzzo W Validating Clustering for Gene Expression Data Bioinformatics 2001 17 309 318 10.1093/bioinformatics/17.4.309 11301299

[B25] Brunet JP Tamayo P Golub T Mesirov J Metagenes and molecular pattern discovery using matrix factorization Proc. of the National Academy of Sciences of the United States of America 2004 101 4164 4169 10.1073/pnas.0308531101 PMC384712 15016911

[B26] Devarajan K Nonnegative Matrix Factorization: An Analytical and Interpretative Tool in Computational Biology PLoS Computational Biology 2008 4 1 12 10.1371/journal.pcbi.1000029 PMC2447881 18654623

[B27] Dudoit S Fridlyand J Bagging to improve the accuracy of a clustering procedure Bioinformatics 2003 19 9 1090 1099 10.1093/bioinformatics/btg038 12801869

[B28] Levine E Domany E Resampling Method for Unsupervised Estimation of Cluster Validity Neural Computation 2001 13 2573 2593 10.1162/089976601753196030 11674852

[B29] Roth V Lange T Braun M Buhmann J A Resampling Approach to Cluster Validation Proc 15th Symposium in Computational Statistics 2002 123 128

[B30] Bertoni A Valentini G Model order selection for bio-molecular data clustering BMC Bioinformatics 2007 8 10.1186/1471-2105-8-S2-S7 17493256 PMC1892076

[B31] Bertrand P Mufti GB Loevinger's measures of rule quality for assessing cluster stability Computational Statistics & Data Analysis 2006 50 992 1015 10.1016/j.csda.2004.10.012

[B32] Klie S Nikoloski Z Selbig J Biological Cluster Evaluation for Gene Function Prediction Journal of Computational Biology 2010 17 1 18 10.1089/cmb.2009.0031 20059365

[B33] Supplementary material web site http://www.math.unipa.it/~raffaele/suppMaterial/speedUp/

[B34] Di Gesú V Giancarlo R Lo Bosco G Raimondi A Scaturro D Genclust: A Genetic Algorithm for Clustering Gene Expression Data BMC Bioinformatics 2005 6 289 10.1186/1471-2105-6-289 16336639 PMC1343581

[B35] Krzanowski W Lai Y A Criterion for Determining the Number of Groups in a Dataset Using Sum of Squares Clustering Biometrics 1985 44 23 34 10.2307/2531893

[B36] Bertoni A Valentini G Randomized maps for assessing the reliability of patients clusters in DNA microarray data analyses Artificial Intelligence in Medicine 2006 37 85 109 10.1016/j.artmed.2006.03.005 16720093

[B37] Salvador S Chan P Determining the number of clusters/segments in hierarchical clustering/segmentation algorithms Tools with Artificial Intelligence, 2004. ICTAI 2004. 16th IEEE International Conference on 2004 576 584 full_text

